# Distributed encoding of curvilinear self-motion across spiral optic flow patterns

**DOI:** 10.1038/s41598-022-16371-4

**Published:** 2022-08-04

**Authors:** Oliver W. Layton, Brett R. Fajen

**Affiliations:** 1 Department of Computer Science, Colby College, Waterville, ME USA; 2 Department of Cognitive Science, Rensselaer Polytechnic Institute, Troy, NY USA

**Keywords:** Neural circuits, Sensory processing, Visual system, Extrastriate cortex, Human behaviour

## Abstract

Self-motion along linear paths without eye movements creates optic flow that radiates from the direction of travel (heading). Optic flow-sensitive neurons in primate brain area MSTd have been linked to linear heading perception, but the neural basis of more general curvilinear self-motion perception is unknown. The optic flow in this case is more complex and depends on the gaze direction and curvature of the path. We investigated the extent to which signals decoded from a neural model of MSTd predict the observer’s curvilinear self-motion. Specifically, we considered the contributions of MSTd-like units that were tuned to radial, spiral, and concentric optic flow patterns in “spiral space”. Self-motion estimates decoded from units tuned to the full set of spiral space patterns were substantially more accurate and precise than those decoded from units tuned to radial expansion. Decoding only from units tuned to spiral subtypes closely approximated the performance of the full model. Only the full decoding model could account for human judgments when path curvature and gaze covaried in self-motion stimuli. The most predictive units exhibited bias in center-of-motion tuning toward the periphery, consistent with neurophysiology and prior modeling. Together, findings support a distributed encoding of curvilinear self-motion across spiral space.

## Introduction

Substantial progress has been made over the past several decades toward understanding how neurons in visual cortex encode information about the environment and state of the observer. The pioneering work of Hubel and Wiesel^1^ exemplifies the successes that have been garnered in primary visual cortex and other areas early in the visual hierarchy^2^. Tuning properties and the function of neurons in areas further along in extrastriate cortex, however, have remained more elusive.

**Figure 1:**
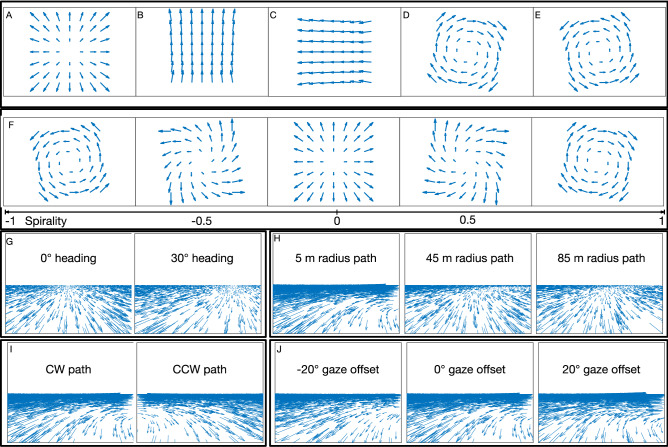
Sample optic flow vector fields and patterns in spiral space continuum. (**A**) Optic flow experienced by an observer moving straight-forward toward a frontoparallel wall. A stationary observer that performs a downward (**B**) or rightward (**C**) eye movement experiences approximately laminar optic flow due to pitch and yaw rotation, respectively. A stationary observer that rotates the head about the line of sight experiences CW (**D**) or CCW (**E**) concentric motion due to roll rotation. (**F**) Sample optic flow patterns in spiral space. The “spirality” metric defines the position in the spiral space and the proportion of roll rotation that is added to the radial translation field (center). Negative (positive) spirality yields CCW (CW) motion. (**G**–**J**) Optic flow fields generated by different types of linear and curvilinear self-motion over a ground plane. (**G**) Self-motion along straight-ahead (left) and rightward (right) linear trajectories. (**H**) Curvilinear self-motion along circular paths with different radii. (**I**) Curvilinear self-motion along CW (left) and CCW (right) 5 m radius circular paths. (**J**) Curvilinear self-motion along 5 m radius circular paths with the observer gaze offset from the path tangent (left: gaze directed outside the future path, center: gaze along path tangent as in (**H**,**I**); right: gaze directed inside the future path).

One such area is the dorsal medial superior temporal area (MSTd), which has long been associated with self-motion perception. Much neurophysiological work has focused on translation, the self-motion scenario wherein the observer moves along a straight path without eye or head movements. Forward translation generates a pattern of motion on the eye (optic flow) that radiates outward from a singularity (radial expansion; Fig. 1A) known as the focus of expansion (FoE), which specifies the observer’s direction of travel (heading)^3^. Many MSTd neurons exhibit tuning to the position of the FoE in the radial optic flow pattern^4,5^ and causal links have been established to heading perception^6–8^.

Translation, together with another type of self-motion known as rotation, fully characterize the possible optic flow patterns (to a first-order approximation) that may arise during movement through a rigid environment^9^. Rotation arises during eye movements and has three primary components: pitch, yaw, and roll. Vertical and horizontal eye movements are examples of pitch and yaw rotation, respectively, and yield mostly uniform motion with some perspective distortion at the periphery (Fig. 1B,C). Clockwise (CW) and counterclockwise (CCW) rotation of the head about the line of sight are examples of roll rotation and generates concentric optic flow patterns (Fig. 1D,E). Combinations of translational and rotational self-motion yield spiral patterns, as Fig. 1F depicts for forward translation and roll. The continuum of patterns has been said to represent a “spiral space”^10,11^. Neurons demonstrate systematic tuning to patterns that span the spiral space in MSTd^10–12^ and in areas 7a^13,14^ and VIP^15^, other areas at a similar depth along the dorsal stream^16^ with extensive optic flow sensitivity.

Much of what is known about the role of these brain areas in self-motion perception presupposes movement along a straight path. The neural basis of more general curvilinear self-motion is presently unknown. In the present study, we performed a computational analysis to determine whether populations of spiral space-tuned neurons could encode self-motion along curvilinear paths. Curvilinear self-motion commonly arises during naturalistic self-motion and yields optic flow (Fig. 1G–J) with both a translational component, due to observer movement, and a rotational component, due to rotation of the view about a point in the world (e.g. center of circular path). In the present study, we examine the case where the observer moves along a circular path of self-motion of constant radius. We focus on the neural encoding of path curvature, path sign, and gaze offset, three key parameters that describe the curvilinear self-motion of the observer. Path curvature is inversely related to the radius of the circular path traversed by the observer (Fig. 1H). Path sign indicates whether the observer moves along a CW or CCW path (Fig. 1I). Gaze offset refers to the horizontal offset in the observer’s gaze (i.e. bias in camera orientation) relative to the straight-ahead (Fig. 1J).

The present study addresses the following questions:

- Could units tuned to optic flow patterns in spiral space accurately encode the curvilinear self-motion of the observer? To what extent does the neural population plausibly account for human perceptual judgments?
- Do contributions predominately come from units tuned to specific patterns or is the model consistent with a distributed representation across spiral space? Are units tuned to radial expansion sufficient to decode accurate curvilinear self-motion estimates?
- What are the singularity tuning characteristics of the units that contribute to estimates of curvilinear self-motion? Specifically, are they uniformly distributed or biased toward the center or periphery?

**Figure 2:**
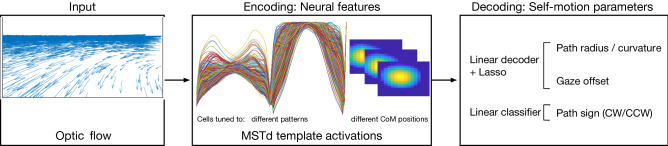
Overview of the neural encoder-decoder system. Optic flow generated by simulated self-motion (left) is passed through a neural model of areas MT and MSTd (center), which contains units tuned to optic flow patterns sampled from spiral space with different preferred centers of motion. The activation of the MSTd units is decoded (right) to recover parameters describing the observer’s curvilinear self-motion (path curvature, gaze offset, and path sign).

To address these questions, we fit linear decoders to predict gaze offset and path curvature parameters from the activations of model MSTd units and compared the accuracy of estimates with human judgments (Fig. 2). We used lasso regularization^17,18^ to create sparse decoding models that select patterns that are most predictive when estimating each self-motion parameter. Models with sparse representations of optic flow capture important properties of MSTd neurons^19^ and human heading perception^20^.

## Results

We simulated a neural model that first processes the input optic flow (e.g. Figs. 1G–J and 2 left panel) with filters, which emulate the direction and speed tuning properties of the medial temporal (MT) area. We then matched the resulting activity distribution with template patterns that represent each unit’s preferred spiral space pattern (Fig. 1F). This feedforward template matching paradigm is widely used to model optic flow selectivity in MSTd^21–26^. We examined the encoding of curvilinear self-motion in a population of model units tuned to optic flow patterns (“MSTd”) sampled from spiral space (henceforth “Full model”). Because one of our datasets consists of optic flow generated by simulated curvilinear self-motion over a ground plane (Fig. 1H–J), the Full model has units tuned to both full-field and hemi-field versions of spiral space patterns, the latter of which only included sensitivity to motion at or below the position of the singularity, which we refer to as the center of motion (CoM). For each preferred optic flow pattern, we simulated units tuned to different CoM positions sampled across the visual field.

We compared the Full model with a simpler model consisting only of units tuned to radial expansion (henceforth “Radial Only model”). We fit the linear decoders associated with the Full and Radial Only MSTd models using the activations produced from 900 optic flow samples that varied with respect to path curvature (radius: 5–200 m), gaze offset (− 35 to + 35°), and path sign (CW, CCW). Path sign was predicted using a linear binary classifier. Unless noted otherwise, simulations involve optic flow from self-motion over a ground plane; we obtained similar results on a dataset consisting of simulated self-motion through a 3D dot cloud (see Supplementary Information).

The lasso yielded sparse models for decoding gaze offset and path curvature that included 758 (3.52%) and 718 (3.34%) units, respectively, of the simulated 21,504 MSTd units derived from the Full model (16 × 16 grid of CoM positions across the visual field tuned to 42 CW and 42 CCW spiral space patterns). The decoders derived from the Radial Only model to estimate gaze offset and path curvature included 83 (32.42%) and 87 (33.98%) units, respectively, of the 256 units (16 × 16 CoM) in the Radial Only model.

**Figure 3:**
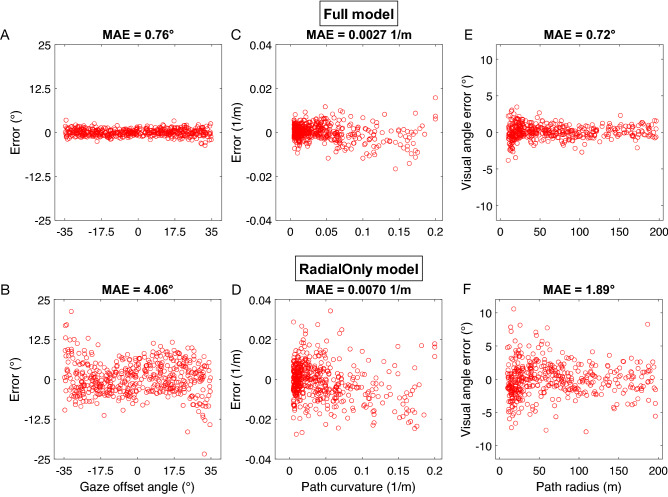
Error in the gaze offset (**A**,**B**) and path curvature (**C**–**F**) estimates produced by the Full model (top row) and Radial Only model (bottom row). (**E**,**F**) Error in path curvature is expressed with respect to the difference in visual angle between the true and predicted path measured at a depth of 10 m. The full model contains units tuned to heterogenous spiral space patterns, while the radial only model contains only units tuned to radial expansion.

Figure 3 summarizes the accuracy of estimates of gaze offset (Fig. 3A,B) and path curvature (Fig. 3C,D) decoded from the full (top row) and radial only (bottom row) models on the 500 novel test optic flow samples. To quantify error across the dataset, we use mean absolute error (MAE). Gaze offset estimates decoded from the Full model (MAE: 0.76°, Fig. 3A) were substantially more accurate than those obtained from the Radial Only model (4.06°, Fig. 3B). Path curvature estimates decoded from the Full model (0.0027 1/m Fig. 3C) were also much more accurate than those obtained from the Radial Only model (0.0070 1/m, Fig. 3D). Although the predictions of the Full model were based on more units than the predictions of the Radial Only model, the Full model achieves more accurate predictions than the Radial Only model even when we constrain it to have the same number of nonzero regression weights (i.e. free parameters). This analysis is described in the Model Comparison section below.

**Figure 4:**
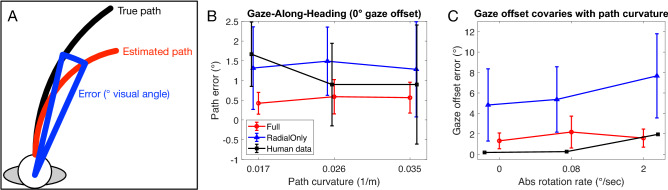
Comparisons between human judgments and estimates produced by the Full and Radial Only decoding models. (**A**) Schematic showing error in path curvature expressed as the angular difference in visual angle subtended by the estimated and true paths (“path error”). (**B**) Comparison between path the error produced by human subjects in the “gaze-along-heading” condition from Li and Cheng^27^ and that produced by the decoding models. In this condition, the gaze offset is 0°. Positive (negative) path error corresponds to an overestimation (underestimation) of path curvature. (**C**) Comparison between the gaze offset error produced by human subjects in the “envelop” condition from Burlingham and Heeger^28^ and that produced by the decoding models. In this condition, gaze offset covaried with path curvature. X values are staggered for visual clarity.

To better appreciate the practical significance of the discrepancy between the models, we expressed the error in path curvature estimates with respect to the difference in visual angle subtended between the true and decoded the paths (“path error”; Fig. 4A). Small angular differences imply close proximity between the two paths from the observer’s vantage point. We measured this difference 10 m from the observer, a distance that has been used to obtain human curvilinear path judgments^27^. This analysis revealed that the Full model estimated paths that deviated by 0.72° (Fig. 3E) compared to 1.89° (Fig. 3F) in the case of the Radial Only model. The Full model correctly classified the path sign of all test samples while the Radial Only model made 35/500 misclassifications (7%).

### Comparison to human judgments

We compared the accuracy of model estimates to that of human judgments of curvilinear self-motion. Our aim was not to perform an exhaustive analysis of the existing psychophysical literature; rather we sought to gauge the sufficiency of model parameter estimates for capturing human performance under the simulated conditions.

We simulated the “gaze-along-heading” condition from Li and Cheng ^27^ in which subjects judged path curvature while viewing simulated movement over a dot-defined ground plane. We used the previously fit decoders to estimate path curvature in this new condition. We expressed decoding error in terms of the previously used difference in visual angle (“path error”; Fig. 4A) measure to afford a direct comparison to the Li and Cheng data. Figure 4B suggests that both models produce human-like estimates when gaze is aligned with the instantaneous heading direction. Consistent with Li and Cheng, the positive error indicates an overestimation of the path curvature in both models. The difference between the path error predicted by the model and the path error based on the human data was significantly smaller for the Full model compared to the Radial Only model [t(59) = 2.25, p = 0.03]. However, inspection of Fig. 4B indicates that the advantage for the Full model is not consistent across path curvatures.

This prompted us to compare performance under more challenging conditions wherein gaze offset varied over a wider range. We simulated the “envelop” condition from Burlingham and Heeger^28^ in which humans judged the gaze offset angle [− 20, 20°] in displays simulating curvilinear self-motion along 43 m and 107 m radius paths. We expressed these path radii with respect to the rotation rate in the optic flow to afford direct comparison to their study (see “Methods”). Figure 4C shows that the Radial Only model does not plausibly account for human gaze offset judgments. The Full model yields comparable error to humans on the high curvature path (2°/s rotation) condition and slightly greater error compared to humans on the straight and 107 m paths, but the discrepancy (1–2°) is quite small. The difference between the gaze offset error predicted by the model and the gaze offset error based on the human data was significantly smaller for the Full model compared to the Radial Only model [t(27) = 5.81, p < 0.001]. This suggests that cells tuned to non-radial patterns in spiral space may play a role in estimating gaze offset with human-like accuracy.

### Model position and pattern tuning characteristics

**Figure 5:**
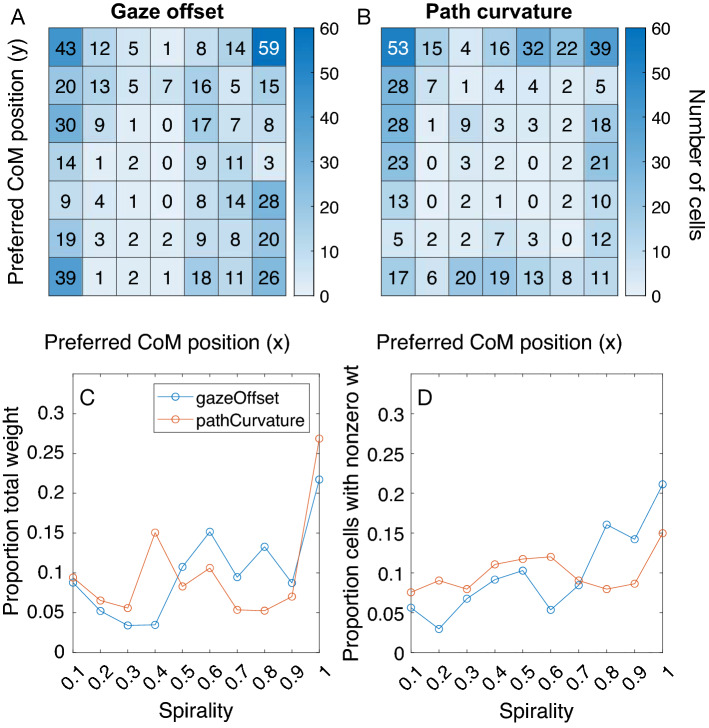
Spatial and pattern tuning properties of gaze offset and path curvature decoding models. (Top row) Histograms showing the number of cells tuned to CoM positions in the different regions of the visual field models included in the gaze offset (**A**) and path curvature (**B**) decoding models. (**C**) The proportion of the total regression weight accounted for by units tuned to different optic flow patterns (spirality) in each decoding model. (**D**) Proportion of cells tuned to different patterns included in each decoding model. Units are grouped in (**C**,**D**) based on the absolute value of spirality.

Given the improved accuracy of the Full model and its consistency with human data, we analyzed the tuning properties of the model units that contribute to self-motion parameter estimates. Figure 5 shows the number of units with different CoM preferences that contribute to gaze offset (Fig. 5A) and path curvature (Fig. 5B) estimates. Far more units tuned to peripheral CoM positions contributed to estimates than those tuned to central CoM positions. That is, the lasso set many more units with central CoM preferences to zero than those with peripheral preferences in both decoding models. As we explain in the Discussion, this finding is compatible with findings from both neurophysiological and computational studies.

Next, we considered how the relative contribution of units varied across pattern within the spiral space. This is quantified in terms of the relative strength of regression weights. Units that share a large proportion of the overall weight make more substantial contribution to parameter estimates than others with a smaller share of the weight. We use the “spirality” metric to express the fraction that the preferred spiral space pattern tuning depends on rotational optic flow. For example, 0 implies sensitivity to radial expansion, ± 1 implies sensitivity to concentric motion, and values in between that approach ± 1 imply sensitivity to spirals with increasing curvature (Fig. 1F). Figure 5C plots the proportion of the total weight that is shared among units tuned to different spiral space patterns in the gaze offset and path curvature decoders. Units tuned to concentric motion accounted for the most weight compared to units tuned to any other pattern type and therefore contribute most to estimates of gaze offset (21.7%; spirality 1) and path curvature (26.9%; spirality 1) in the Full model. The remaining proportion of the weight in both decoders is broadly shared across the other pattern types. This suggests a distributed representation across spiral space, which agrees with the findings of Xu and colleagues that show fairly even contributions among neurons tuned across spiral space toward monkey heading judgments^10^.

Figure 5D shows the proportion of units tuned to each pattern type included in each decoder. Taken together with Fig. 5C, units tuned to concentric motion represent the most numerous group and contribute most to estimates compared to any other single group.

### Model comparison

The simulation results from Figs. 3 and 4 reveal the insufficiency of a model constructed with units tuned only to radial patterns. However, findings depicted in Fig. 5C raise the possibility that units tuned to other narrow ranges of the spiral space could primarily mediate the estimates produced by the Full model. We examined this possibility further by constructing models that constrain decoding from focused portions of spiral space. Specifically, we compared the accuracy of gaze offset and path curvature estimates garnered by the Full model with separate decoders fit to units tuned only to radial expansion (‘Radial Only’); to spirals associated with the lowest, middle, and upper third of spiralities; and to concentric motion (‘Concentric Only’). We also sought to address the extent to which the Full model’s superior accuracy to the Radial Only model may be attributed to the larger number of units that it includes. To that end, we included in the model comparison a version of the Full model constrained to possess the same number of nonzero weights as the Radial Only model (‘Full Limited’).

**Figure 6:**
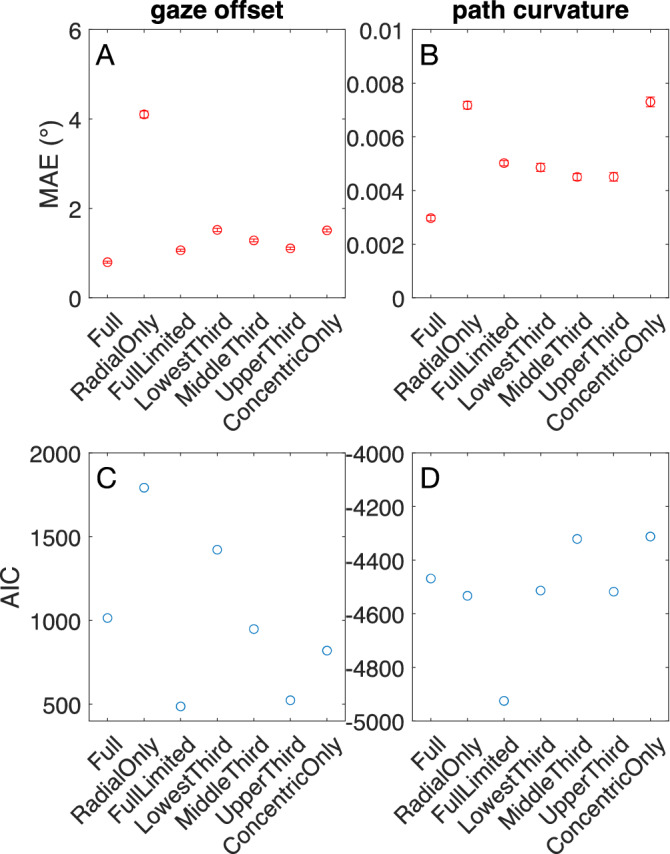
Comparison of decoding models. Top row: The MAE for gaze offset (**A**) and path curvature (**B**) parameter estimates. Bottom row: AIC computed for each of the models. In addition to the Full and Radial Only models, we considered a version of the Full model constrained to have the same number of nonzero weights as the Radial Only model (i.e. same number of free parameters; Full Limited). The Lowest Third, Middle Third, and Upper Third correspond to models that consist only of units tuned to lowest third (nearly radial), middle third, and upper third (nearly concentric) of spiralities in the spiral space. Concentric Only indicates a model that consists only of concentric units. Error bars correspond to ± 1 standard deviation across MAE estimates obtained over 50 bootstraps. The small error indicates robustness to the specific set of optic flow stimuli included in the fitting process of each decoding model.

Consistent with the analysis shown in Fig. 3 and Fig. 6 shows that the Radial Only model garnered considerably worse accuracy than the Full model when estimating gaze offset and path curvature. Decoding from any one group of spiral-tuned units or the concentric units produced gaze offset estimates that were almost as accurate as those produced by the Full model (Fig. 6A). In the case of path curvature, the accuracy garnered by the spiral-tuned decoders was approximately halfway between that achieved by the Radial Only and Full models (Fig. 6B). In contrast to gaze offset, the accuracy of the path curvature estimates decoded from the concentric units was poor and similar to that decoded from the Radial Only model. The discrepancy in performance between concentric unit models could stem from the larger number of concentric motion units that contribute to gaze offset predictions (Fig. 5D). It is possible that sensitivity to concentric motion on its own may not capture enough information that is essential for predicting path curvature across a range of gaze offsets (Fig. 1J).

The accuracy of estimates produced by the Full Limited model was in between those achieved from Radial Only and Full models and comparable to the models constructed from thirds of the spiral space. The improved accuracy of the Full Limited model compared to the Radial Only model suggests an advantage of decoding gaze offset and path curvature from neurons tuned to a broad, distributed set of spiral patterns, even when controlling for the number of free parameters.

We examined the tradeoff between free parameters and accuracy more formally by computing the Akaike Information Criterion (AIC) for each model (Fig. 6C,D). This analysis also helps contextualize the accuracy of the other models, each of which contained different numbers of nonzero weights. For gaze offset, the numbers of nonzero weights were 385, 243, 83, and 73 for the Lowest Third, Middle Third, Upper Third, and Concentric Only models, respectively. For path curvature, these numbers were 268, 407, 342, and 171 for the Lowest Third, Middle Third, Upper Third, and Concentric Only models, respectively. Figure 6C,D reveals that the Full Limited model is favored according to the AIC metric for both gaze offset and path curvature estimates. Taken together, these results support a distributed representation of curvilinear self-motion across spiral space.

## Discussion

While neurophysiology has linked the perception of linear self-motion to the activity of neurons in MSTd, the neural basis of more general curvilinear self-motion perception has been unclear. Our computational analysis suggests that tuning to combinations of translational and rotational optic flow in spiral space could support estimates of curvilinear self-motion. Decoding from units tuned to radial motion resulted in substantially less accurate and more variable estimates than the full spiral space model. While radial units in our simulations accounted for human judgments when gaze was aligned with the straight-ahead, they did not when gaze offset covaried with path curvature. The full spiral space model accounted for human judgments in both scenarios. Several of our analyses indicated a distributed representation of gaze offset and path curvature across spiral space. We found considerable redundancy in the representation of gaze offset—decoding from units tuned to non-radial patterns in spiral space yielded estimates that closely approximately those obtained by the Full model. This was mostly true of path curvature, albeit to a weaker extent and decoding from units tuned to concentric motion alone yielded poor estimates that were similar to those produced by the radial subpopulation. Overall, our findings support the possibility that MSTd could jointly signal the observer’s linear and curvilinear self-motion.

We used lasso regularization to create sparse linear decoding models that select the units that are most predictive of each self-motion parameter. In the resulting models, we found an overrepresentation of units tuned to peripheral centers of motion (Fig. 5) regardless of whether the optic flow simulated self-motion both over a ground plane and through a 3D dot cloud (see Supplementary Information). This agrees with the peripheral bias in the distribution of MSTd heading preferences reported by neurophysiological studies^4,29^. Peripheral bias in the population tuning increases sensitivity to central headings^30^ and enhances the overall accuracy of heading estimates^25^. Both outcomes may arise because broad, Gaussian-like tuning curves of MSTd neurons tuned to peripheral headings change most rapidly for central headings^30,31^. Our model is compatible with this hypothesis since the MSTd units are center-weighted about the preferred singularity position (Eq. 16). Beyeler and colleagues have proposed a computational model that explains many physiological properties of MSTd neurons, including the population peripheral bias, as an emergent outcome of a dimensionality reduction process^19^. The model produces a sparse representation of optic flow fields encountered during random combinations of translation and rotation. It is interesting that both their model and ours yield peripheral bias in CoM selectivity despite the difference in approach. The lasso regularization used in the present study also favors a model fit with a sparse set of predictors. This converging finding further supports the idea that peripheral bias in CoM selectivity may result from a process that promotes a sparse distributed representation of optic flow. It is noteworthy that the peripheral bias was found despite uniformity in the parameters used to generate the stimuli and regardless of whether the range of gaze offsets was restricted to ± 35 deg (in the present study) or spanned the entire 360° range (in Beyeler et al.). The relationship between the input statistics, tuning curving properties, and peripheral bias should be explored further in future research.

The use of a linear decoder in the present study also advances neural modeling of optic flow processing by expanding the range of self-motion properties that can be estimated. Within the literature on this topic^21,23,24,32–35^, which spans almost 30 years, the standard approach to “reading out” the model’s heading estimate is to find the focus of expansion (FoE) location of the most active MSTd unit. This approach makes sense for pure translational self-motion because there is a simple one-to-one correspondence between self-motion direction and FoE location. However, when we expand the scope to include complex self-motion trajectories and the full gamut of MSTd cells types, it no longer makes sense to assume that the model’s best estimate of self-motion is reflected in the activity of a single unit. A more effective approach is to look at the global pattern of activity across units to extract self-motion estimates. The approach adopted in the present study offers a way to decode multiple self-motion parameters (e.g., gaze angle, path curvature) from the global activity across cells tuned to radial, spiral, and concentric flow patterns.

We emphasize that although neurons in MSTd, VIP, and 7a demonstrate spiral space pattern selectivity, no existing neurophysiological study has specifically linked this tuning to curvilinear self-motion perception. Therefore, it remains an open question whether these cortical areas possess specialized mechanisms that subserve curvilinear path perception. Cheng and Gu have shed some light on this issue, demonstrating that neurons in MSTd, VIP, and VPS exhibit tuning to translation, rotation, and the combinations thereof that arise during curvilinear movement on a 6 DOF platform. Interestingly, the analysis performed by Cheng and Gu revealed a distributed representation of translation and rotation, which supports our findings. The conditions in this study involve vestibular stimulation without accompanying optic flow, so it is unclear how these cells might relate to spiral space optic flow tuning. Nevertheless, the robust vestibular tuning and ~ 80% linear decoder accuracy suggests that vestibular signals could contribute significantly toward the neural basis of curvilinear self-motion perception when the information is available. Further neurophysiological work is required to clarify the connection between spiral space and vestibular tuning.

It should be noted that the present study does not address the influence of eye rotation, which have been shown to modulate MSTd signals during self-motion^36–38^ and human self-motion judgments^39–41^. Eye rotations introduce an endogenous source of rotation into the optic flow field that is independent from the rotation caused by curvilinear self-motion. To estimate curvilinear self-motion during eye movements, the prevalent efference copy theory and related models propose that the visual system relies on nonvisual signals (e.g. proprioceptive or motor signals specifying eye rotation rate) to factor out the rotational flow due to eye movements, leaving on the rotational flow due curvilinear self-motion^42,43^. Such a mechanism could be used to remove the effects of eye rotation prior to decoding from our model. However, the extent to which MSTd decomposes the optic flow field into distinct translational and rotational components visually^44^ or in the presence of non-visual signals^36^ has been questioned. The mechanism by which the visual system tolerates rotation in optic flow during self-motion remains unclear.

Some have examined linear heading perception when gaze remains fixed and eye movements are simulated within the virtual environment of the display (“simulated eye movement condition”)^39^. In the absence of proprioceptive or motor signals that accompany real eye movements and when the scene lacks dense motion parallax^45^, humans appear to treat the simulated rotation as curvilinear self-motion without eye movements^46,47^. This suggests that rotation due to curvilinear self-motion is not factored out, decomposed, or removed, at least for the purposes of estimating curvilinear self-motion^48^. The system presented here is consistent with this proposal, since neither the neural nor the decoding models explicitly remove rotation due to curvilinear self-motion. Indeed, pattern tuning in MSTd may emerge through the dimensionality reduction that neurons perform on their motion inputs^19^ rather than a process whose purpose is to segment translation from rotation.

## Methods

### Optic flow datasets

We created an optic flow dataset consisting of 10 frame digital sequences of simulated curvilinear self-motion over a ground plane (2000 dots) at 64 × 64 pixel resolution. In each video, the simulated observer translated at 3 m/s along a circular path. The virtual camera had a 90° horizontal and vertical field of view and a height of 1.61 m above the ground. On each video frame, we computed the optic flow using a pinhole camera model^49^ and standard analytic equations^9^. We clipped and replaced dots that exited the field of view or valid relative depth range (1–50 m) to ensure that the same number of dots always remained visible. Figure 1G–J shows some example optic flow fields from the dataset.

### Training sets

The training set used to fit the decoders consisted of 900 videos. We sampled the gaze offset parameter on a regular grid to ensure balanced coverage (− 35 to 35° in steps of 8.7°). We sampled path radius differently than the other parameters due to the nonlinear relationship between circular path radius and curvature (Eq. 1). We accounted for this by sampling small radii more finely than large radii using the following recursive generating formula:

1 $${\mathrm{r}}_{\mathrm{next}}=1.078{\mathrm{r}}_{\mathrm{prev}}.$$

We used a starting radius of 5 m and selected the coefficient so that Eq. (1) would generate 50 radii between 5 and 200 m. We doubled the number of radii by including both CW and CCW versions of each path.

### Test sets

We generated a 500-video test set to assess the quality of predictions made by the fit decoders. Gaze offset was sampled from a uniform distribution [− 35, 35°]. We used kernel density estimation (KDE) to randomly sampled path radii in proportion to the training set values. As in the training set, this biased values toward smaller radii that produce larger variation in path curvature. We fit the path radii used in the training set to an empirical probability density function via the MATLAB function *ksdensity*, which we then sampled within the training set range with 0.1 m granularity. As in the training set, we also included CCW versions of these paths.

For Fig. 4, we generated optic flow sequences that emulated the gaze-along-heading condition from Li and Cheng^45^: a gaze offset of 0° and 28, 38, and 58 m path radii. We simulated 20 repetitions of each path radius condition across which only the random placement of dots varied. The Li and Cheng test set consisted of 60 videos.

### 3D dot cloud environment

For the simulations described in Supporting Information, we used a dataset that satisfies the same train and test set parameters, except that we distributed the 2000 dots in a 3D cloud rather than on a ground plane. These simulations were the basis for our comparison with the Burlingham and Heeger^28^ data. Burlingham and Heeger used simulated curvilinear self-motion with 1.5 m/s translation and 0, 0.8, or 2.0°/s rotation. The latter two curvilinear cases correspond to path radii of 107 m and 43 m, respectively. We compared corresponding human judgments of gaze offset with model predictions obtained on the 3D cloud test set stimuli that had a path radius within 2.5 m of the radii used by Burlingham and Heeger. To make a comparison to the straight path condition (0°/s rotation), we considered the predictions made for test stimuli with path radii ≥ 180 m. For such large path radii, the influence of rotation is small (~ 0.45 $^\circ$/s rotation) and the optic flow appears effectively radial.

### Decoding

We fit a linear model with lasso regularization to the neural activations produced by the neural model (described below) at the end of each video (frame 10) in the optic flow training set. The models for decoding path curvature and gaze offset made use of the *lasso* MATLAB function. We set the regularization parameter such that the mean squared error (MSE) of the fit was within 1 standard error of the minimum MSE computed with fivefold cross validation. To fit the Full Limited model, we used the *DFMax* argument of the *lasso* function to constrain the number of nonzero weights in the fit to the those from the Radial Only model. We classified path sign according to a support vector machine binary classifier using the *fitcsvm* MATLAB function configured with the default linear kernel.

The values depicted in Fig. 6A,B reflect the outcome of bootstrap procedure performed 50 times. For each bootstrap, we simulated each model with optic flow sampled with replacement from the original dataset while preserving the total number of samples. Figure 6A,B shows the MAE and its standard deviation computed over each set of 50 bootstrap estimates.

### Neural model of MT and MSTd

**Figure 7:**
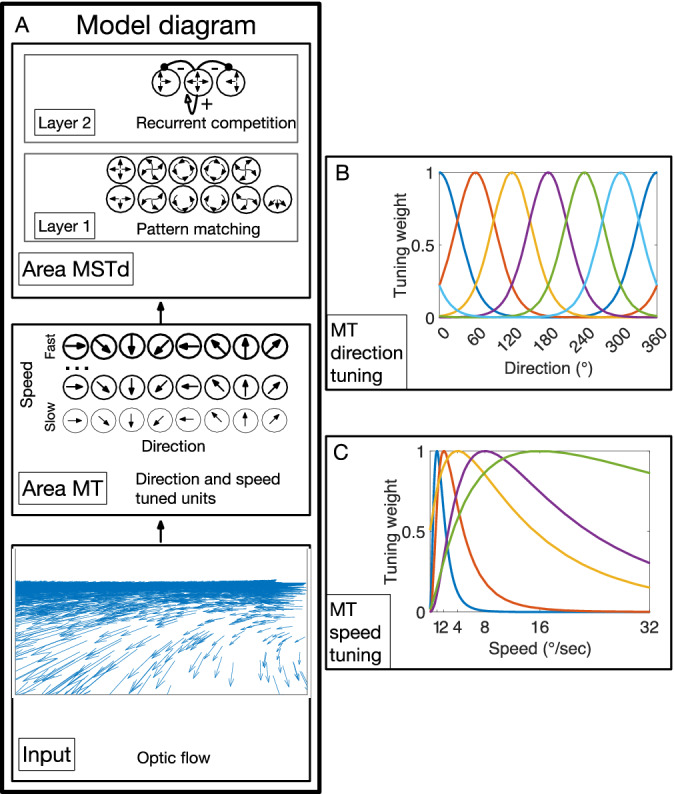
Overview of the Competitive Dynamics neural model of MT-MSTd. (**A**) Model area MT contains direction and speed tuned units. Units in model area MSTd integrate motion signals from MT consistent with the preferred motion pattern and compete with one another. MSTd units are tuned to full and hemi-field motion in a pattern continuum: radial, CCW spirals, CCW centers, CW spirals, CW centers, and ground flow. (**B**) Example MT direction tuning curves. (**C**) Example MT speed tuning curves.

We processed the optic flow datasets with the Competitive Dynamics (CD) neural model of MT and MSTd (Fig. 7)^22,35,50,51^. Populations of model neurons that emulate a range of physiologically supported properties transform the optic flow into neural signals from which we subsequently decode the parameters describing the observer’s self-motion. The CD model accounts for human judgments of self-motion, including in the presence of independently moving objects^22,52^, and object motion during self-motion^50,51,53^. We made several key changes to the most recent version of the model^50,51^:

- Given focus in the present work on self-motion estimation, we only simulated the requisite MT^+^–MSTd pathway of the model, excluding the MT^-^–MSTv object motion pathway^50,51^.
- We simulated units tuned to direction and speed only, omitting disparity tuning.
- Each MSTd unit received input from a random subset of the MT^+^ units^20^. In prior versions of the model, MSTd units were fully connected to the constellation of MT^+^ units that compose the preferred pattern.
- For greater clarity and to better manage the greater diversity of cell types simulated (e.g. radial, spiral, concentric), we divided model MSTd into several processing stages (layers). The first stage integrates the match between each optic flow template over time. This is followed by spatial smoothing of the activation of units tuned to similar CoMs and patterns. Finally, units compete with one another in an on-center/off-surround contrast-enhancing network.

These changes and the complete model details will be fully described in the following sections. Figure 7 summarizes the architecture of the CD model simulated here and Table 1 specifies the values of parameters used to simulate the neural model.

**Table 1:** Parameters used in the neural model.

Parameter	Description	Value
${\mathrm{N}}^{\mathrm{s}}$	Number of preferred speeds	5
${\upsigma }_{\mathrm{v}}$	Speed tuning bandwidth	Normal($\upmu =1.16^\circ ,\upsigma =0.5^\circ$)
${\mathrm{s}}_{0}$	Non-negative offset parameter to prevent singularity in speed logarithm	Exp($\uplambda =0.25^\circ /\mathrm{s}$)
${\mathrm{v}}_{\mathrm{pref}}$	Preferred speed of MT neuron	$\mathrm{U}\left(0.5, 2.0\right)^\circ /\mathrm{s}$, $\mathrm{U}\left(2.0, 4.3\right)^\circ /\mathrm{s}$, $\mathrm{U}\left(4.3, 7.6\right)^\circ /\mathrm{s}$, $\mathrm{U}\left(\mathrm{7.6,12.7}\right)^\circ /\mathrm{s}$, $\mathrm{U}\left(12.7, 32.0\right)^\circ /\mathrm{s}$
${\mathrm{N}}^{\mathrm{d}}$	Number of preferred directions	24
–	Shape of MT^+^ speed layers	64 × 64 × 24 × 5
$\upeta$	Von Mises direction tuning curve bandwidth parameter	3°
${\mathrm{\alpha }}^{\mathrm{MT}}$	MT^+^ passive decay rate	1
${\uptau }^{\mathrm{MT}}$	Depressing synapse time constant	0.1
${\upkappa }^{\mathrm{MT}}$	Rate at which the synaptic efficacy decreases	10
${\mathrm{N}}^{\mathrm{MSTd},\mathrm{com}}$	Number of units tuned to CoM positions for each motion pattern (radial, spiral, etc.) arranged in regular across the visual field	16 × 16 = 256
${\overrightarrow{\uplambda }}^{\mathrm{spl}}$	Spirality of patterns to which spiral cells are tuned. Negative (positive) values produce CCW (CW) spirals	± [0.0, 0.05, …, 1.0]
${\mathrm{N}}^{\mathrm{spl}}$	Number of CW and CCW spiral patterns to which units are tuned	21 (CW) + 21 (CCW) = 42
–	Indices of units in pattern tuning continuum. Units corresponding to the endpoints exhibit tuning to radial expansion or CW/CCW center patterns	CCW full-field spiral (decreasing spirality): 1–21 CW full-field spiral (increasing spirality): 22–42 CW hemi-field spiral (decreasing spirality): 43–63 CCW hemi-field spiral (increasing spirality): 64–84
–	Shape of each MSTd layer	16 × 16 × 84
${\mathrm{N}}^{\mathrm{T}}$	Number of MT^+^ connections within each MSTd pattern template	200
${\mathrm{b}}^{\mathrm{MSTd}}$	Rate at which MT^+^ connection weights decrease with distance from the preferred CoM position	1e−3
${\mathrm{\alpha }}^{\mathrm{MSTd},1\mathrm{a}}$	Layer 1a unit passive decay rate	1
${\overrightarrow{\upsigma }}^{\mathrm{MSTd},1\mathrm{b},\mathrm{com}}$	Layer 1b Gaussian 2D spatial smoothing kernel horizontal and vertical standard deviations	[5, 5]
${\mathrm{r}}^{\mathrm{MSTd},1\mathrm{b},\mathrm{com}}$	Diameter of Layer 1b Gaussian 2D spatial smoothing kernel	8
${\upsigma }^{\mathrm{MSTd},1\mathrm{b},\mathrm{pat}}$	Layer 1b Gaussian 1D pattern smoothing standard deviation	1.5
${\mathrm{r}}^{\mathrm{MSTd},1\mathrm{b},\mathrm{pat}}$	Diameter of Layer 1b Gaussian 1D pattern smoothing kernel	7
${\mathrm{\alpha }}^{\mathrm{MSTd},1\mathrm{b}}$	Layer 1b unit passive decay rate	1
${\mathrm{\alpha }}^{\mathrm{MSTd},\mathrm{c}}$	Layer 2 net input adaptive threshold passive decay rate	1
${\mathrm{\alpha }}^{\mathrm{MSTd},2}$	Layer 2 unit passive decay rate	10
${\upbeta }^{\mathrm{MSTd},2}$	Layer 2 unit excitatory upper bound	3

### Model area MT

We simulated the population of direction and speed tuned neurons in MT with excitatory surrounds (MT^+^), which project to MSTd^16,54,55^ and influence self-motion signals therein^56^.

MT^+^ unit receptive fields (RFs) tuned to like speeds and directions were arranged in a regular 64 × 64 spatial grid across the visual field.

### MT speed tuning

We simulated units tuned to 5 $({\mathrm{N}}^{\mathrm{s}})$ different preferred speeds. Each unit’s speed tuning followed a log-normal distribution:

2 $$\mathrm{S}\left({\mathrm{s}}_{\mathrm{x},\mathrm{y}};{\mathrm{v}}_{\mathrm{pref}},{\mathrm{s}}_{0},{\upsigma }_{\mathrm{v}}\right)=\mathrm{exp}\left(-\frac{{\mathrm{log}\left(\frac{{\mathrm{s}}_{\mathrm{x},\mathrm{y}}+{\mathrm{s}}_{0}}{{\mathrm{v}}_{\mathrm{pref}}+{\mathrm{s}}_{0}}\right)}^{2}}{2{\upsigma }_{\mathrm{v}}^{2}}\right),$$

where ${\upsigma }_{\mathrm{v}}$ defines the speed tuning bandwidth; ${\mathrm{s}}_{0}$ defines a non-negative offset parameter to prevent the singularity in the logarithm at 0; and ${\mathrm{v}}_{\mathrm{pref}}$ defines the preferred speed of the model neuron. Given that MT neuron speed tuning varies considerably, we sampled values from probability distributions that approximate neurophysiological fits to these parameters. Based on Fig. 4 of Ref.^57^, we sampled ${\upsigma }_{\mathrm{v}}$ from a Gaussian distribution (mean: 1.16°, SD: 0.5°) and ${\mathrm{s}}_{0}$ from an exponential distribution (λ: 0.25°/s). Consistent with Fig. 8 of Nover et al., we sampled ${\mathrm{v}}_{\mathrm{pref}}$ from five uniform distributions with endpoints that yielded octave spaced bins (see Table 1).

We computed optic flow speed at retinotopic position (x, y) as:

3 $${\mathrm{s}}_{\mathrm{x},\mathrm{y}}= \sqrt{{\mathrm{u}}_{\mathrm{x},\mathrm{y}}^{2}+ {\mathrm{v}}_{\mathrm{x},\mathrm{y}}^{2},}$$

where ${\mathrm{u}}_{\mathrm{x},\mathrm{y}}$ and ${\mathrm{v}}_{\mathrm{x},\mathrm{y}}$ represent the horizontal and vertical components of the optic flow vector.

### MT direction tuning

We simulated units tuned to 24 $({\mathrm{N}}^{\mathrm{d}})$ motion directions (Fig. 7). We modeled each unit’s direction tuning width using the following von Mises distribution

4 $$\mathrm{V}\left(\mathrm{d};{\upmu }_{\mathrm{dir}},\upeta \right)=\frac{{\mathrm{e}}^{\mathrm{\eta cos}\left(\mathrm{d}-{\upmu }_{\mathrm{dir}}\right)}}{{\mathrm{e}}^{\upeta }},$$

where $\mathrm{d}$ represents the direction of the optic flow in the receptive field, ${\upmu }_{\mathrm{dir}}$ represents the unit’s preferred direction, the shape parameter $\upeta$ controls the direction tuning width. We set $\upeta =3^\circ$ to approximate the 90° full-width at half-maximum bandwidth of MT neurons^6,19^. We computed the direction of the optic flow vector ${\overrightarrow{\mathrm{v}}}_{\mathrm{x},\mathrm{y}}=({\mathrm{u}}_{\mathrm{x},\mathrm{y}},{\mathrm{v}}_{\mathrm{x},\mathrm{y}})$ at position (x, y) via the two-argument form of the arctangent function:

5 $${\Theta }_{\mathrm{x},\mathrm{y}}= {\text{atan2}}\left({\mathrm{v}}_{\mathrm{x},\mathrm{y}}, {\mathrm{u}}_{\mathrm{x},\mathrm{y}}\right).$$

### MT unit activation

The net input to the MT^+^ unit tuned to speed $\mathrm{s}$, direction $\mathrm{d}$, and has its RF center at position $(\mathrm{x},\mathrm{ y})$ is the product of the directional $\mathrm{V}\left({\Theta }_{\mathrm{x},\mathrm{y}};\mathrm{d},\upeta \right)$ and speed $\mathrm{S}\left({\mathrm{s}}_{\mathrm{x},\mathrm{y}};\mathrm{s},{\mathrm{s}}_{0},{\upsigma }_{\mathrm{v}}\right)$ tuning curve outputs:

6 $${\mathrm{I}}_{\mathrm{s},\mathrm{x},\mathrm{y},\mathrm{d}}^{\mathrm{MT}}=\mathrm{V}\left({\Theta }_{\mathrm{x},\mathrm{y}};\mathrm{d},\upeta \right)\mathrm{S}\left({\mathrm{s}}_{\mathrm{x},\mathrm{y}};\mathrm{s},{\mathrm{s}}_{0},{\upsigma }_{\mathrm{v}}\right).$$

We modeled the dynamics of MT^+^ unit ${\mathrm{m}}_{\mathrm{s},\mathrm{x},\mathrm{y},\mathrm{d}}$ as a simple leaky integrator:

7 $$\frac{{\mathrm{dm}}_{\mathrm{s},\mathrm{x},\mathrm{y},\mathrm{d}}}{\mathrm{dt}}=-{\mathrm{\alpha }}^{\mathrm{MT}}{\mathrm{m}}_{\mathrm{s},\mathrm{x},\mathrm{y},\mathrm{d}}+\left(1-{\mathrm{m}}_{\mathrm{s},\mathrm{x},\mathrm{y},\mathrm{d}}\right){\mathrm{I}}_{\mathrm{s},\mathrm{x},\mathrm{y},\mathrm{d}}^{\mathrm{MT}},$$

where ${\mathrm{\alpha }}^{\mathrm{MT}}$ is the passive decay rate of the cell.

### MT output

Among models that explain MSTd responses based on their feedforward input from MT, those that include a nonlinearity that compresses MT signals perform best^26^. The compressive nonlinearity could be explained by synaptic depression, the tendency for the same inputs to lose their efficacy over time. We modeled MT^+^ synaptic depression ${\mathrm{h}}_{\mathrm{s},\mathrm{x},\mathrm{y},\mathrm{d}}$ as follows:

8 $$\frac{1}{{\uptau }^{\mathrm{MT}}}\frac{{\mathrm{dh}}_{\mathrm{s},\mathrm{x},\mathrm{y},\mathrm{d}}}{\mathrm{dt}}=1-{\mathrm{h}}_{\mathrm{s},\mathrm{x},\mathrm{y},\mathrm{d}}\left(1+{\upkappa }^{\mathrm{MT}}{\mathrm{h}}_{\mathrm{s},\mathrm{x},\mathrm{y},\mathrm{d}}\right),$$

9 $${\mathrm{O}}_{\mathrm{s},\mathrm{x},\mathrm{y},\mathrm{d}}={\mathrm{h}}_{\mathrm{s},\mathrm{x},\mathrm{y},\mathrm{d}}{\mathrm{m}}_{\mathrm{s},\mathrm{x},\mathrm{y},\mathrm{d}},$$

where ${\mathrm{O}}_{\mathrm{s},\mathrm{x},\mathrm{y},\mathrm{d}}$ denotes the output signal from MT^+^ units to MSTd, ${\uptau }^{\mathrm{MT}}$ is the synaptic time constant, and ${\upkappa }^{\mathrm{MT}}$ represents the rate at which the efficacy of the input signal ${\mathrm{m}}_{\mathrm{s},\mathrm{x},\mathrm{y},\mathrm{d}}$ declines over time^58^.

### MSTd net input

We simulated MSTd cells tuned to radial expansion ${(\overrightarrow{\Lambda }}_{\mathrm{rad},\mathrm{i},\mathrm{j},\mathrm{x},\mathrm{y}})$ and spiral ${(\overrightarrow{\Lambda }}_{\mathrm{spl},\mathrm{i},\mathrm{j},\mathrm{x},\mathrm{y}})$ optic flow patterns that have been used to characterize MSTd selectivity in neurophysiological studies^5,11^.

10 $${\overrightarrow{\Lambda }}_{\mathrm{rad},\mathrm{i},\mathrm{j},\mathrm{x},\mathrm{y}}=\left({\mathrm{v}}_{\mathrm{rad},\mathrm{dx}},{\mathrm{v}}_{\mathrm{rad},\mathrm{dy}}\right)=\left(\mathrm{x}-\mathrm{i}\right),\left(\mathrm{y}-\mathrm{j}\right),$$

11 $${\overrightarrow{\Lambda }}_{\mathrm{cir},\mathrm{i},\mathrm{j},\mathrm{x},\mathrm{y}}=\left({\mathrm{v}}_{\mathrm{cir},\mathrm{dx}},{\mathrm{v}}_{\mathrm{cir},\mathrm{dy}}\right)=\left(\mathrm{y}-\mathrm{j}\right),-\left(\mathrm{x}-\mathrm{i}\right),$$

12 $${\overrightarrow{\Lambda }}_{\mathrm{spl},\mathrm{i},\mathrm{j},\mathrm{x},\mathrm{y}}=\left({\mathrm{v}}_{\mathrm{spl},\mathrm{dx}},{\mathrm{v}}_{\mathrm{spl},\mathrm{dy}}\right)=\left(1-{\uplambda }^{\mathrm{spl}}\right){\overrightarrow{\Lambda }}_{\mathrm{rad},\mathrm{i},\mathrm{j},\mathrm{x},\mathrm{y}}+{\uplambda }^{\mathrm{spl}}{\upzeta }^{\mathrm{spl}}{\overrightarrow{\Lambda }}_{\mathrm{cir},\mathrm{i},\mathrm{j},\mathrm{x},\mathrm{y}}.$$

In Eqs. (10–12), $(\mathrm{i},\mathrm{ j})$ indicates the location of the center of motion (CoM), which corresponds to the FoE in the case of radial expansion; $(\mathrm{x},\mathrm{y})$ indicates visuotopic position of the local motion vector, ${\upzeta }^{\mathrm{spl}}$ is 1 for CW spirals and − 1 for CCW spirals; and ${\uplambda }^{\mathrm{spl}}$ is a scalar selected in the range 0–1 that represents the spirality of the pattern. Setting ${\uplambda }^{\mathrm{spl}} =0$ produces a radial expansion flow field, ${\uplambda }^{\mathrm{spl}} =1$ produces a circular center flow field $({\overrightarrow{\Lambda }}_{\mathrm{cir},\mathrm{i},\mathrm{j},\mathrm{x},\mathrm{y}})$, and $0<{\uplambda }^{\mathrm{spl}} <1$ creates spiral patterns spanning a continuum between these two extremes. We created MSTd units tuned to ${\mathrm{N}}^{\mathrm{spl}}$ spiral patterns by sampling regularly spaced values of ${\uplambda }^{\mathrm{spl}}$ (see Table 1). We simulated MSTd units tuned to both full-field and lower the hemifields. The latter was achieved by removing motion vectors above the CoM.

We implemented MSTd tuning to the preferred pattern using direction templates that select MT^+^ signals when they appear in appropriate spatial locations. For example, in the case of a cell tuned to radial expansion with a centrally positioned FoE, the rightward direction template pools the responses of MT + cells tuned to rightward motion when their receptive fields coincide with the right side of the visual field. The following equations define the RF template ${\mathrm{T}}_{\uppsi ,\mathrm{d},\mathrm{i},\mathrm{j},\mathrm{x},\mathrm{y}}$ for pattern $\uppsi$ that integrates MT^+^ cells tuned to direction $\mathrm{d}$, normalized by the total number of pooled cells $(\widehat{\mathrm{x}}\widehat{\mathrm{y}})$:

13 $${\upchi }_{\uppsi ,\mathrm{d},\mathrm{i},\mathrm{j},\mathrm{x},\mathrm{y}}=\mathrm{atan}2\left({\Lambda }_{\uppsi ,\mathrm{i},\mathrm{j},\mathrm{x},\mathrm{y},\mathrm{dy}},{\Lambda }_{\uppsi ,\mathrm{i},\mathrm{j},\mathrm{x},\mathrm{y},\mathrm{dx}}\right),$$

14 $${\widehat{\mathrm{T}}}_{\uppsi ,\mathrm{d},\mathrm{i},\mathrm{j},\mathrm{x},\mathrm{y}}=\left\{\begin{array}{cc}1& \frac{2\uppi (\mathrm{d}-1-\widehat{\mathrm{d}} )}{\widehat{\mathrm{d}}}\\ 0& \mathrm{otherwise}\end{array}\right.<{\upchi }_{\uppsi ,\mathrm{d},\mathrm{i},\mathrm{j},\mathrm{x},\mathrm{y}}<\frac{2\uppi (\mathrm{d}-\widehat{\mathrm{d}})}{\widehat{\mathrm{d}}},$$

15 $${\text{T}}_{{{{\uppsi }},{\text{d}},{\text{i}},{\text{j}},{\text{x}},{\text{y}}}} = \frac{1}{{\widehat{{\text{x}}}{{\widehat{{\text{y}}}}} }}\widehat{{\text{T}}}_{{{{\uppsi }},{\text{d}},{\text{i}},{\text{j}},{\text{x}},{\text{y}}}} .$$

In Eq. (14), $\widehat{\mathrm{d}}$ indexes the ${\mathrm{N}}^{\mathrm{d}}$ MT preferred directions.

Within each pattern template, we sampled a small subset of the possible connections from MT^+^ units (e.g. 200 instead of $64\times 64\times 24=98304$). Sparse connectivity is more biologically plausible and substantially improves the algorithmic efficiency of the model. We implemented this by randomly selecting only ${\mathrm{N}}^{\mathrm{T}}$ connections to MT^+^ units within each template tuned to pattern $\uppsi$ with CoM positioned at $(\mathrm{i},\mathrm{j})$. After drawing ${\mathrm{N}}^{\mathrm{T}}$ pairs of $(\mathrm{x},\mathrm{y})$ indices per template, we selected directions at each spatial location according to the uniform distribution $\mathrm{U}[1, {\mathrm{N}}^{\mathrm{d}}]$. We denote the set of ${\mathrm{N}}^{\mathrm{T}}$ three-tuple $(\mathrm{x},\mathrm{y},\mathrm{d})$ indices of MT^+^ units sampled within each template $\Omega$. We repeated this process for each of the 5 $({\mathrm{N}}^{\mathrm{s}})$ preferred speeds. This transformed the original $64\times 64\times 24\times 5$ signal into one with shape ${\mathrm{N}}^{\mathrm{d}}\times {\mathrm{N}}^{\mathrm{T}}$.

The following equation matches the direction templates with the output signals from MT^+^ ${(\mathrm{O}}_{\mathrm{s},\mathrm{x},\mathrm{y},\mathrm{d}})$ at the set of sampled spatial and directional indices $(\mathrm{x},\mathrm{y},\mathrm{d})\in\Omega$ to compute the net input $({\mathrm{R}}_{\uppsi ,\mathrm{i},\mathrm{j}})$ to the MSTd unit that prefers the pattern $\uppsi$ with a CoM positioned at $(\mathrm{i},\mathrm{j})$:

16 $${\mathrm{R}}_{\uppsi ,\mathrm{i},\mathrm{j}}=\frac{1}{{\mathrm{N}}^{\mathrm{T}}}{\sum }_{\mathrm{s}}{\sum }_{\left(\mathrm{x},\mathrm{y},\mathrm{d}\right)\in\Omega }{\mathrm{e}}^{-{\mathrm{b}}^{\mathrm{MSTd}}({\left(\mathrm{x}-\mathrm{i}\right)}^{2}+{\left(\mathrm{y}-\mathrm{j}\right)}^{2})}\frac{{\mathrm{T}}_{\uppsi ,\mathrm{d},\mathrm{i},\mathrm{j},\mathrm{x},\mathrm{y}}{\mathrm{O}}_{\mathrm{s},\mathrm{x},\mathrm{y},\mathrm{d}}}{\underset{\mathrm{d}}{\mathrm{max}}{\mathrm{O}}_{\mathrm{s},\mathrm{x},\mathrm{y},\mathrm{d}}}.$$

In Eq. (16), the exponential function makes MSTd units more sensitive to motion nearby the preferred CoM position and the parameter ${\mathrm{b}}^{\mathrm{MSTd}}$ modulates how sensitivity decreases with distance^22,32,35^. Dividing by the maximum directional input at each position ensures that dominant inputs make comparable contributions across visuotopic space before the exponential distance weighting from the CoM is applied.

### MSTd layer 1a

We arranged MSTd units into separate layers for organizational clarity. Note that these layers do not correspond to anatomical laminae. Layer 1 corresponds to MSTd units that are driven by their pattern tuning and perform spatiotemporal averaging of their input ${\mathrm{R}}_{\uppsi ,\mathrm{i},\mathrm{j}}$. We divided the temporal (Layer 1a) and spatial (Layer 1b) averaging into two sequential stages. Layer 2 includes recurrent competitive connections that normalize the total activation while enhancing the contrast between strongly and weakly active MSTd units. We rely on the signals produced by Layer 2 for decoding the parameters specifying the observer’s self-motion.

Dynamics in Layer 1a average each MSTd unit’s net input (Eq. 16) over time:

17 $$\frac{{\mathrm{da}}_{\uppsi ,\mathrm{i},\mathrm{j}}}{\mathrm{dt}}=-{\mathrm{\alpha }}^{\mathrm{MSTd},1\mathrm{a}}{\mathrm{a}}_{\uppsi ,\mathrm{i},\mathrm{j}}+\left(1-{\mathrm{a}}_{\uppsi ,\mathrm{i},\mathrm{j}}\right){\mathrm{R}}_{\uppsi ,\mathrm{i},\mathrm{j}}.$$

### MSTd layer 1b

Units in MSTd Layer 1b pool the activation among units tuned to similar CoM locations and patterns along the spiral continuum (Fig. 1F). We achieved this by convolving the spatial grid of signals emanating from Layer 1a units with a two-dimensional Gaussian ${\mathrm{G}}_{2}(\mathrm{x},\mathrm{y};\overrightarrow{\upmu },\overrightarrow{\upsigma })$

18 $${\mathrm{G}}_{2}\left(\mathrm{x},\mathrm{y};\overrightarrow{\upmu },\overrightarrow{\upsigma }\right)=\frac{1}{2\uppi {\upsigma }_{\mathrm{x}}{\upsigma }_{\mathrm{y}}}{\mathrm{e}}^{-\left({\left(\frac{\mathrm{x}-{\upmu }_{\mathrm{x}}}{\sqrt{2}{\upsigma }_{\mathrm{x}}}\right)}^{2}+{\left(\frac{\mathrm{y}-{\upmu }_{\mathrm{y}}}{\sqrt{2}{\upsigma }_{\mathrm{y}}}\right)}^{2}\right)},$$

19 $${\mathrm{I}}_{\uppsi ,\mathrm{i},\mathrm{j}}^{\mathrm{MSTd},1\mathrm{b}}={\sum }_{\mathrm{n}}{\sum }_{\mathrm{m}}{\mathrm{G}}_{2}(\mathrm{n},\mathrm{m};\left(\mathrm{i},\mathrm{j}\right),{\overrightarrow{\upsigma }}^{\mathrm{MSTd},1\mathrm{b},\mathrm{com}}{)\mathrm{a}}_{\uppsi ,\mathrm{i}-\mathrm{n},\mathrm{j}-\mathrm{m}},$$

and convolving the pattern signals at each CoM position with a one-dimensional Gaussian:

20 $${\mathrm{G}}_{1}\left(\mathrm{x};\upmu ,\upsigma \right)=\frac{1}{\sqrt{2\uppi }\upsigma }{{\mathrm{e}}^{-\left(\frac{\mathrm{x}-\upmu }{\sqrt{2}\upsigma }\right)}}^{2},$$

21 $${\mathrm{J}}_{\uppsi ,\mathrm{i},\mathrm{j}}^{\mathrm{MSTd},1\mathrm{b}}={\sum }_{\mathrm{n}}{\mathrm{G}}_{1}\left(\mathrm{n};\uppsi ,{\upsigma }^{\mathrm{MSTd},1\mathrm{b},\mathrm{pat}}\right){\mathrm{I}}_{\uppsi -\mathrm{n},\mathrm{i},\mathrm{j}}^{\mathrm{MSTd},1\mathrm{b}}.$$

Because we arranged units tuned to similar patterns along a circular spiral pattern continuum (see Table 1), we wrapped the convolution in Eq. (21) around the boundaries.

The dynamics of Layer 1b units obey:

22 $$\frac{{\mathrm{db}}_{\uppsi ,\mathrm{i},\mathrm{j}}}{\mathrm{dt}}=-{\mathrm{\alpha }}^{\mathrm{MSTd},1\mathrm{b}}{\mathrm{b}}_{\uppsi ,\mathrm{u},\mathrm{j}}+\left(1-{\mathrm{b}}_{\uppsi ,\mathrm{i},\mathrm{j}}\right){\mathrm{J}}_{\uppsi ,\mathrm{i},\mathrm{j}}^{\mathrm{MSTd},1\mathrm{b}}.$$

### MSTd layer 2

The input to Layer 2 undergoes an adaptive threshold to suppress signals weaker than the mean activation value ${\mathrm{c}}_{\uppsi ,\mathrm{i},\mathrm{j}}$ achieved among units tuned to the same pattern over the recent time history

23 $$\frac{{\mathrm{dc}}_{\uppsi }}{\mathrm{dt}}=-{\mathrm{\alpha }}^{\mathrm{MSTd},\mathrm{c}}{\mathrm{c}}_{\uppsi }+\left(1-{\mathrm{c}}_{\uppsi }\right)\left(\frac{1}{{\mathrm{N}}^{\mathrm{MSTd},\mathrm{com}}}{\sum }_{\mathrm{m}}{\sum }_{\mathrm{n}}{\mathrm{b}}_{\uppsi ,\mathrm{m},\mathrm{n}}\right),$$

where ${\mathrm{N}}^{\mathrm{MSTd},\mathrm{com}}$ represents the total number of units tuned to the same pattern with differing CoM positions. The net input to Layer 2 is the Layer 1b output signal with the threshold applied:

24 $${\mathrm{I}}_{\uppsi ,\mathrm{i},\mathrm{j}}^{\mathrm{MSTd},2}={\lceil{\mathrm{b}}_{\uppsi ,\mathrm{i},\mathrm{j}}-{\mathrm{c}}_{\uppsi }\rceil}^{+},$$

where ${\lceil\cdot \rceil}^{+}$ represents the half-wave rectification $\mathrm{max}(\cdot ,0)$.

Layer 2 integrates signals from Layer 1b and contains recurrent connections within the layer that normalize activation, facilitate competition, and stabilize signals under changing conditions^22^. Units obey the recurrent competitive field with a contrast-enhancing feedback transfer function $\mathrm{f}\left(\mathrm{w}\right)={\mathrm{w}}^{2}$, where $\mathrm{w}$ represent the unit activation^59^:

25 $$\frac{{\mathrm{dz}}_{\uppsi ,\mathrm{i},\mathrm{j}}}{\mathrm{dt}}=-{\mathrm{\alpha }}^{\mathrm{MSTd},2}{\mathrm{z}}_{\uppsi ,\mathrm{i},\mathrm{j}}+\left({\upbeta }^{\mathrm{MSTd},2}-{\mathrm{z}}_{\uppsi ,\mathrm{i},\mathrm{j}}\right)\left(\mathrm{f}\left({\mathrm{z}}_{\uppsi ,\mathrm{i},\mathrm{j}}\right)+{\mathrm{D}}_{\uppsi ,\mathrm{i},\mathrm{j}}\mathrm{E}\right)-{\mathrm{z}}_{\uppsi ,\mathrm{i},\mathrm{j}}\left({\sum }_{\mathrm{m}\ne \mathrm{i}}{\sum }_{\mathrm{n}\ne \mathrm{j}}\mathrm{f}\left({\mathrm{z}}_{\uppsi ,\mathrm{m},\mathrm{n}}\right)+{\sum }_{\mathrm{p}\ne\uppsi }{\sum }_{\mathrm{m}\ne \mathrm{i}}{\sum }_{\mathrm{n}\ne \mathrm{j}}{\mathrm{D}}_{\mathrm{p},\mathrm{m},\mathrm{n}}\right).$$

In Eq. (25), ${\upbeta }^{\mathrm{MSTd},2}$ represents the excitatory upper bound of each unit and the terms involving $\mathrm{f}(\cdot )$ implement competition among units tuned to the same pattern. The terms ${\mathrm{D}}_{\uppsi ,\mathrm{i},\mathrm{j}}\mathrm{E}$ and ${\sum }_{\mathrm{p}\ne\uppsi }{\sum }_{\mathrm{m}\ne \mathrm{i}}{\sum }_{\mathrm{n}\ne \mathrm{j}}{\mathrm{D}}_{\mathrm{p},\mathrm{m},\mathrm{n}}$ describe excitatory and inhibitory recurrent connections, respectively, that subserve activity normalization across the layer^60^. In the former term, ${\mathrm{D}}_{\uppsi ,\mathrm{i},\mathrm{j}}$ denotes the difference between each unit’s current activation and the feedforward input signal:

26 $${\mathrm{D}}_{\uppsi ,\mathrm{i},\mathrm{j}}={\lceil{\mathrm{z}}_{\uppsi ,\mathrm{i},\mathrm{j}}-{\mathrm{I}}_{\uppsi ,\mathrm{i},\mathrm{j}}^{\mathrm{MSTd},2}\rceil}^{+}.$$

The variable $\mathrm{E}$ defines the discrepancy between input and activation across the entire layer:

27 $$\mathrm{E}={\sum }_{\mathrm{p}}{\sum }_{\mathrm{m}}{\sum }_{\mathrm{n}}{\mathrm{D}}_{\mathrm{p},\mathrm{m},\mathrm{n}}.$$

This means that the network attempts to conserve the total activation across the entire layer, not just among units tuned to the same pattern.

## Supplementary Information

Supplementary Information.

## Data Availability

Competitive Dynamics model activations and the decoding model code are available on Github at https://github.com/owlayton/R-Curvilinear-Path-Encoding. The ground optic flow dataset is available on OSF, https://osf.io/su8h3/?view_only=0a18371f0828412f91895512a6656ea4.
