# Supplementary material for: Distributed encoding of curvilinear self-motion across spiral optic flow patterns
Source: Sci Rep. 2022 Aug 4;12:13393. doi: 10.1038/s41598-022-16371-4 (PMC9352735; doi:10.1038/s41598-022-16371-4)
Supplement: Supplementary file 1 — Supplementary Information. [file 41598_2022_16371_MOESM1_ESM.pdf]

Supplementary information

Here we present simulations of the same scenarios used in the main text, but with a 3D dot cloud instead of a ground environment. The only difference in the dataset is that we distributed the 2000 dots in a 3D cloud rather than on a ground plane. Overall, we found that estimates were less accurate, but the results are qualitatively similar to those obtained with the ground environment. We attribute the decrease in accuracy to the fact that during curvilinear self-motion, dots in similar regions of the visual field may move in different directions depending on their depth. This kind if motion transparency does not occur with a ground plane, where dots move consistently and do not occlude one another over time.

The lasso yielded sparse models for decoding gaze offset and path curvature that included 1751 (8.14%) and 1547 (7.19%) units, respectively, of the simulated 21,504 MSTd units derived from the Full model (16x16 grid of CoM positions across the visual field tuned to 42 CW and 42 CCW spiral space patterns). The decoders derived from the Radial Only model to estimate gaze offset and path curvature included 144 (56.25%) and 7 (2.73%) units, respectively, of the 256 units (16x16 CoM) in the Radial Only model.

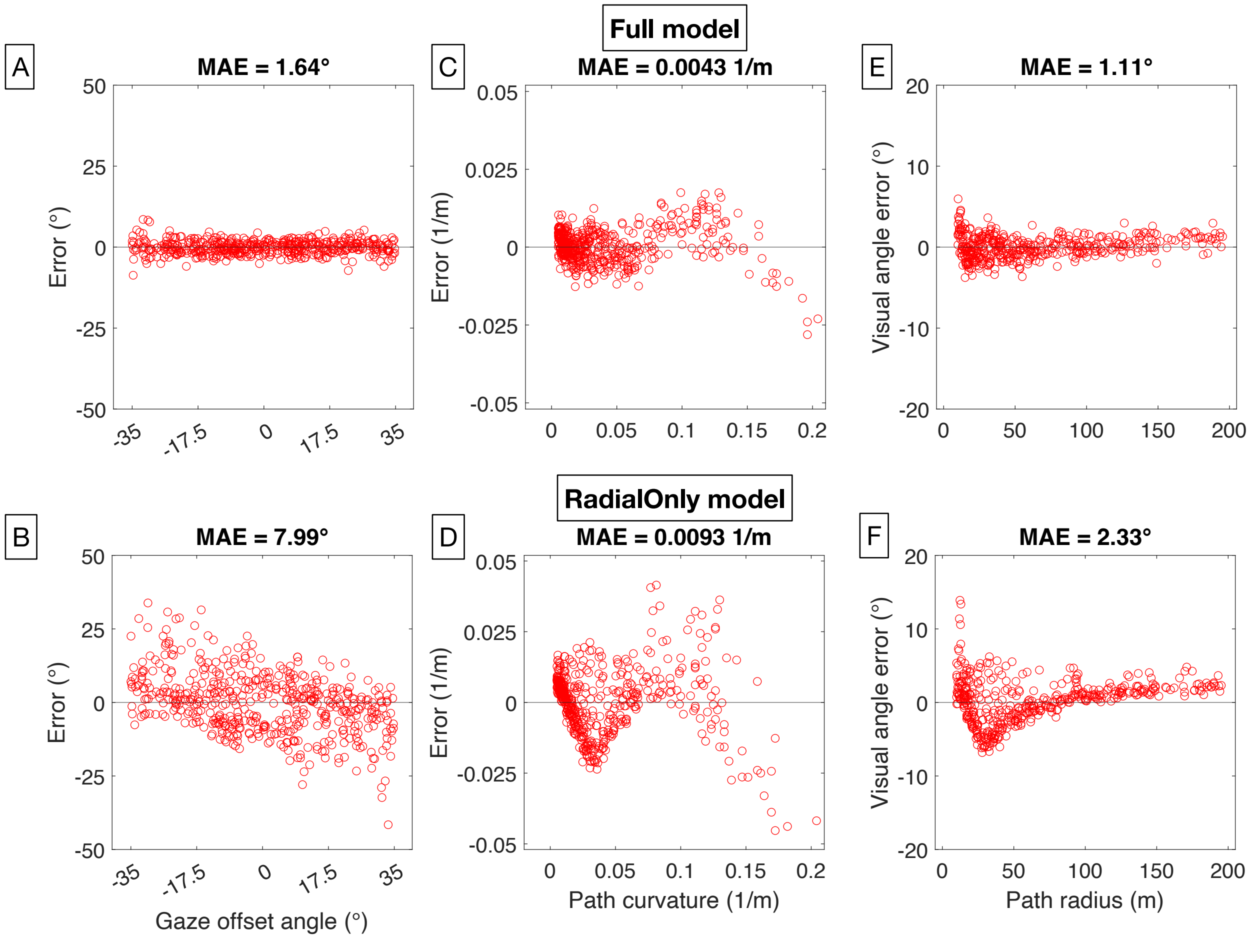

Figure S1: Error in the gaze offset (a,b) and path curvature (c-f) estimates produced by the Full model (top row) and Radial Only model (bottom row). (e,f) Error in path curvature is expressed with respect to the difference in visual angle between the true and predicted path measured at a depth of 10 m.

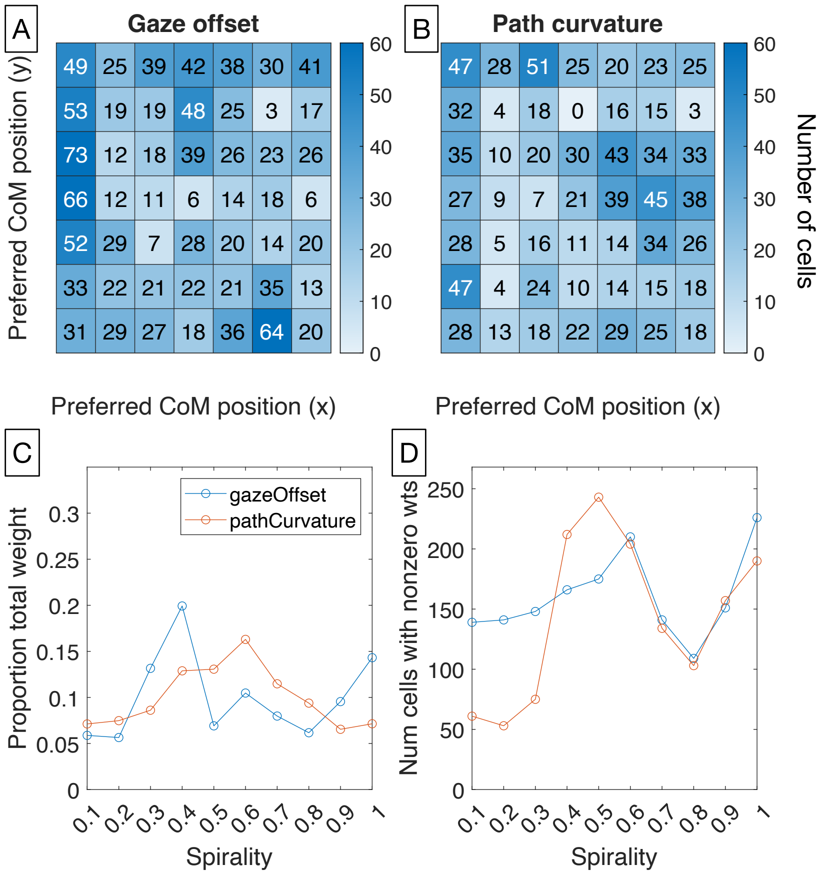

Figure S2: Spatial and pattern tuning properties of gaze offset and path curvature decoding models. (top row) Histograms showing the number of cells tuned to CoM positions in the different regions of the visual field models included in the gaze offset (a) and path curvature (b) decoding models. (c) The proportion of the total regression weight accounted for by units tuned to different optic flow patterns (spirality) in each decoding model. (d) Number of cells tuned to different patterns included in each decoding model. Units are grouped in (c,d) based on the absolute value of spirality.

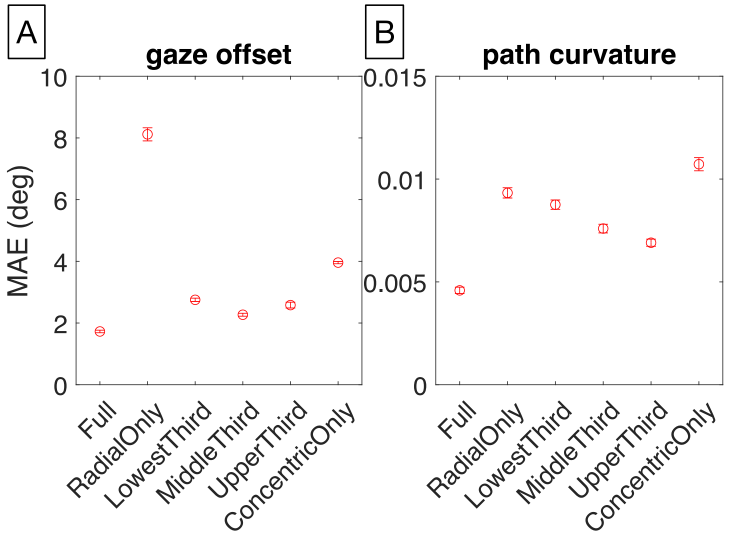

Figure S3: The MAE for parameter estimates. These statistics reflect estimates over 50 bootstraps. The small error bars indicate robustness to the specific set of optic flow stimuli included in the fitting process of each decoding model.
